# Removal of a Large Scrotal Tumor

**Published:** 1847-12

**Authors:** Lewis A. Sayre

**Affiliations:** New York


Art. IV.—*Removal of a Large Scrotal Tumor.* By Lewis A. Sayre, M.D., of New York.

The subject of the following operation was a Spaniard, 21 years of age, who had had hydrocele from his fifth year. The hydrocele continued to enlarge until he attained his nineteenth year, when I operated upon him, and drew off nearly a quart of water from each side, injected iodine, and the hydrocele was completely cured. But the dartos muscle had been so long distended that it became paralyzed, and consequently the scrotum remained pendulous, dragging down the integuments of the abdomen so as completely to sheath and conceal the penis. The mass formed a tumor between the thighs as large as a child's head—measuring, in fact, twelve inches from the base of the penis to its lower edge near the anus.

The danger of this operation, as is known, is in the secondary hemorrhage. When the vessels are first divided, they retract so that it is often impossible to secure them; and after the wound has been dressed a few hours, numberless small vessels pour out blood into the sac, and cause it to burst, or else establish a formidable suppuration. I therefore had made two pieces of bent steel, twelve inches long, with a screw in each end, and placing one on each side of the scrotum parallel with the raphe, screwed the two together, with the tumor between them, sufficiently tight to cut off the circulation in the part I wished to excise. I then transfixed the mass with eight needles, an inch or more apart, so as to catch the septum and lining serous membrane; I then excised the mass with a single sweep of the knife. No hemorrhage whatever followed. I proceeded next to place over the needles the twisted suture, as for hare-lip, and took an interrupted suture between each of these, which brought the parts accurately in apposition. Finally I unscrewed the clamps (I know not what else to call them) a little, so as not to interrupt the circulation entirely in the lips of the wound, but still kept them sufficiently tight to prevent any hemorrhage into the sac. In five days the whole wound, twelve inches in length, had healed by the first intention, not one drop of matter having been formed.

The advantages of this mode are very obvious. Surgeons are well aware of the difficulty of accurately adjusting wounds of the scrotum. Adhesive plaster is useless; and the dartos muscle is so constantly in motion that we cannot keep the parts in constant apposition, even with closely applied sutures; whereas by the apparatus I employed we keep the parts accurately in place, and by the control of the screws we may keep hemorrhage completely in check, at the same time that it may be so regulated as not to interfere with the healthy circulation of the parts.

New York, November 1, 1847.

